# Using long-range freeze-preventive vaccine carriers in Nepal: A study of equipment performance, acceptability, systems fit, and cost

**DOI:** 10.1016/j.jvacx.2022.100146

**Published:** 2022-02-11

**Authors:** Sandeep Kumar, Pat Lennon, Nancy Muller, Surendra Uranw, Mercy Mvundura, Alexandra Sibole, Steven Diesburg, Joe Little, Arindam Ray, Jhalak Sharma Gautam, Rupa Rajbhandari Singh, Nilambar Jha

**Affiliations:** a PATH, New Delhi, India; b PATH, Seattle, WA, USA; c B.P. Koirala Institute of Health Sciences, Dharan, Nepal; d Bill & Melinda Gates Foundation, India Country Office, New Delhi, India; e Government of Nepal, Family Health Division, Ministry of Health and Population, Kathmandu, Nepal

**Keywords:** AHW, auxiliary health worker, ANM, auxiliary nurse midwife, BPKIHS, B.P. Koirala Institute of Health Sciences, CCH, cold chain handler, FPVC, freeze-preventive vaccine carrier, HP, health post, MKT, mean kinetic temperature, MOHP, Ministry of Health and Population, N/A, not applicable, PQS, Performance, Quality and Safety, SVC, standard vaccine carrier, VVM, vaccine vial monitor, WHO, World Health Organization, Vaccine cold chain, Cold chain equipment, Freeze-preventive vaccine carrier, Immunization, Innovation, Vaccine freezing

## Abstract

Preventing vaccine freezing is one of the biggest challenges in vaccine management. Until 2018, vaccine carriers used in the immunization program lacked features to prevent vaccine freezing. Freeze-preventive vaccine carriers (FPVCs) have an engineered liner that buffers vaccines from direct exposure to frozen ice packs. A field evaluation of three FPVCs was conducted in 24 health posts in eastern Nepal. The objective was to evaluate the FPVCs’ performance, acceptability, systems fit, and cost, to inform prequalification and introduction planning. The study was carried out in two phases: in the first phase, FPVCs containing dummy vaccines (labeled “Not for Human Use”) were transported to outreach sessions along with a standard vaccine carrier (SVC); in the second phase, the FPVCs were used for transporting vaccines taken to outreach sessions and used for vaccinating eligible children. The study gathered quantitative and qualitative data from health workers, logbooks, and electronic temperature monitors placed inside and outside the FPVCs. Results indicate the FPVCs successfully prevented temperatures below 0 °C more than 99% of the time—except at one site, where ambient temperatures were below the minimum rated testing temperature specified by the World Health Organization. Internal cool-down times for the FPVCs were highly variable, as were mean kinetic temperatures, possibly driven by the wide range of ambient temperatures and higher-than-expected variations in freezer performance, which, along with the need to transport ice packs to some locations, affected ice-pack temperatures. Almost all health workers requested smaller, lighter-weight FPVCs but appreciated the FPVCs’ ability to prevent vaccines from freezing while avoiding undue heat exposure. FPVCs had benefit-cost ratios greater than 1 and hence good value for money. Results point to the importance of understanding the intended environment of use and the need for smaller, short-range as well as long-range carriers.

## Highlights

• Freeze-preventive vaccine carriers successfully prevent vaccine freezing.
• The technology was shown to be cost effective.
• Both short-range and long-range freeze-preventive carriers are needed.
• Weight and size were a concern for many health workers.

## Introduction

Preventing vaccine freezing remains one of the biggest barriers in vaccine management [1], [2]. There is currently no vial-level indicator of freeze exposure for vaccines and so health workers worldwide have only the shake test [3], [4] as a means to verify whether vaccine freezing has occurred—and anecdotal reports indicate this test is not often done [5], [6]. Unlike heat exposure, where the effect on potency takes from 2 to 30 days at 37 °C, freezing can happen in an instant for freeze-sensitive vaccines [7]. Freezing can irreversibly damage vaccines adsorbed onto aluminum salt adjuvants, such as diphtheria, tetanus, pertussis, hepatitis B, and *Haemophilus influenzae* type b, reducing vaccine potency and compromising protective immunogenicity in recipients. A number of studies conducted in countries of all income levels have reported the exposure of vaccines to freezing temperatures during storage and transport in the vaccine cold chain [8], especially in standard vaccine carriers (SVCs) at lower levels of the health system [8]. Studies have also indicated that the World Health Organization (WHO)-recommended practice of either conditioning ice packs or using cool water packs is not routinely followed [2], [8]. To address the freezing of vaccines during outreach, the WHO Performance, Quality and Safety (PQS) team developed new performance specifications for freeze-preventive vaccine carriers (FPVCs) [9]. FPVCs have an engineered liner that buffers vaccines from direct exposure to frozen ice packs.

To protect vaccines, FPVCs and SVCs must maintain internal temperatures of 0 °C to +10 °C, the accepted temperature range for passively cooled devices, which is slightly different from actively cooled refrigerators (+2 °C to +8 °C). The primary advantage of FPVCs is in preserving the potency of freeze-sensitive vaccines, even when frozen-solid (unconditioned) ice packs are used. FPVCs are also expected to simplify logistics by reducing the amount of time required by cold chain handlers (CCHs) to prepare the carriers for outreach sessions. An additional advantage could include reduction in training burden required to effectively use cool water packs or conditioned ice packs in SVCs.

This study evaluated three laboratory-tested and conditionally prequalified long-range FPVCs—produced by AOV International, Leff Trade, and Blowkings—in two districts of eastern Nepal. B.P. Koirala Institute of Health Sciences (BPKIHS) in Dharan, Nepal, in collaboration with PATH, conducted the field evaluation in 24 health posts (HPs). The primary study objectives were to evaluate the performance, acceptability, systems fit, and cost of the FPVCs within an existing immunization system.

## Materials and methods

### Country and site selection

Nepal was selected as the location of the field evaluation due to the strong relationship between the Nepal Ministry of Health and Population (MOHP) and PATH, and due to the country’s variety of climatic and environmental conditions. The study was conducted in two types of terrain (hilly and plains) to evaluate the FPVCs in a variety of climatic and environmental conditions. There had to be an alternate delivery mechanism to transport vaccines to HPs. In total, 24 HPs were purposively selected in conjunction with the MOHP: 12 in two areas (called blocks) of Dhankuta District (hilly region) and 12 in three blocks of Sunsari District (plains region). All phases of the study were conducted in the same 24 HPs.

### Study design

The evaluation was conducted in two phases: phase 1 (mid-May through August 2018), phase 1a (early May through early July 2019), and phase 2 (mid-November through December 2019). The Nepal MOHP gave approval to BPKIHS for each phase of the study. The BPKIHS Institutional Review Committee and National Health Research Council provided ethical review and approval. In phase 1, the study team evaluated the AOV FPVC (and for comparison, paired SVCs); in phase 1a, the Leff Trade and Blowkings FPVCs (and paired SVCs); and in phase 2, all three FPVC types (no SVCs). In phases 1 and 1a, the FPVCs contained dummy vaccines labeled with an “X” and “Not for Human Use.” The FPVCs were transported to routine immunization outreach sessions along with the SVCs, and were opened for set periods of time to simulate actual use (i.e., the lid was removed three times per outreach session for 1 min at a time). The MOHP then reviewed the data and gave approval to proceed with phase 2. In phase 2, the FPVCs were used to transport real vaccines for actual use; no SVCs were used.

Twelve of the HPs had a refrigerator for storing vaccines and were able to freeze ice packs on-site. The HPs without freezers obtained ice packs from a nearby cold chain point (facility that stores vaccines) or a district vaccine store. Half of the HPs were instructed to condition ice packs prior to loading them into the vaccine carriers; the other half, not to condition ice packs. To condition the ice packs, CCHs left the ice packs outside the freezers for 30 to 60 min (based on room temperature) or until the ice was slushy when shaken; unconditioned ice packs were considered frozen solid.

In phase 1, the study evaluated 24 AOV FPVCs (one for each HP); in phase 1a, 12 Leff Trade and 12 Blowkings FPVCs; and in phase 2, seven AOV, nine Leff Trade, and eight Blowkings FPVCs. Of the seven HPs that used AOV, three were directed to use frozen-solid ice packs, while four were directed to use conditioned ice packs; of the nine HPs that used Leff Trade, five used frozen solid, four used conditioned; and of the eight HPs that used Blowkings, four used frozen solid and four used conditioned.

Data collection in each phase occurred in two rounds corresponding to the monthly immunization schedules of the study regions. CCHs prepared the FPVCs, which were transported to session sites by vaccinators such as auxiliary health workers (AHWs) and auxiliary nurse midwives (ANMs). The AHWs and ANMs ensured placement of two temperature monitoring devices (detailed in section 2.4) inside and outside the FPVCs. The devices automatically recorded the temperature every 10 min, while the AHWs and ANMs manually recorded session start and end times in logbooks. The study team used the resulting data to calculate mean internal and mean kinetic temperature (MKT) inside the vaccine compartment, frequency and extent of temperature excursions, effect of ambient temperature on internal temperature, cool-down times and rates, and percentage vaccine vial monitor (VVM) life loss. The quantities of vaccines taken to each session were also recorded in the logbooks and used to calculate the benefit-cost ratio of the FPVCs. In addition, the temperatures of some of the freezers were collected to understand the environment in which the ice packs were frozen.

Using a structured in-depth interview guide, an experienced qualitative researcher collected qualitative data on the acceptability of the FPVCs among ANMs, AHWs, and CCHs at the end of each phase (in this paper, referred to collectively as “health workers”). Interviews were conducted with 12 health workers at the end of phase 1 and 24 health workers at the end of phases 1a and 2. Discussions focused on how well the FPVCs performed during outreach, including storage capacity, freeze prevention, size and weight, and staffing- and training-related aspects. Similar feedback was collected from medical officers and district immunization officers.

### FPVC equipment design

**Fig. 1:**
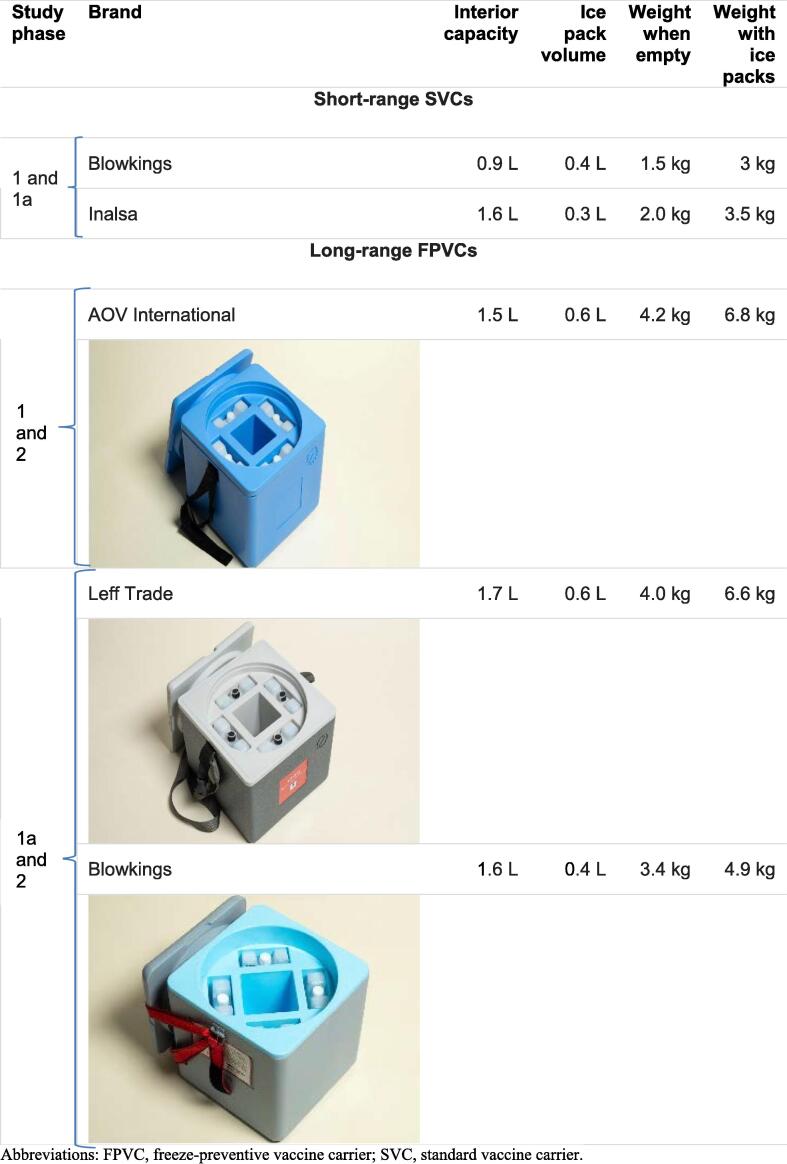
Short- and long-range vaccine carriers used in the study.

All the FPVCs used in this study have an engineered liner that buffers vaccines from direct exposure to frozen ice packs. To prevent reaching freezing temperatures from the rapid cooling that occurs when ice packs are placed inside an SVC, FPVCs cool more slowly. To address the slower cool-down rate, WHO requires verification testing confirming that it takes no longer than 8 h for the FPVC storage compartment to cool from an ambient temperature of +43 °C to +10 °C or lower after placing the ice packs inside [9]. WHO specifies cold life (temperatures maintained between 0 °C and +10 °C) for FPVCs must be at least 15 h for short range and 30 h for long range when tested at 43 °C ambient [9]. All FPVCs must weigh no more than 8.0 kg when fully loaded. Fig. 1 shows the relevant specifications of the carriers used in this study.

### Temperature recording devices

#### LogTag

The study used LogTag® TRIX-8 electronic temperature monitors to gauge internal and ambient temperatures. These devices are WHO PQS prequalified and are a well-accepted global standard for reliable data collection in refrigerators. The LogTag recorders were placed in freezers in 17 of the 24 HPs (7 sites were excluded as they did not have freezers) and both inside and outside the SVCs and FPVCs at all 24 sites. Block monitors downloaded the temperature data monthly for analysis.

#### Parsyl

A new electronic temperature monitoring device, the Parsyl Trek 1.1, was also included, to ascertain its ability to record temperature and humidity in field conditions and to rapidly upload data. The Trek 1.1 is commercially available but not PQS prequalified. The humidity and light data were used for study analysis; the temperature data were not used to evaluate carrier performance.

### Training

Before each phase, all staff involved in routine immunization at the 24 HPs were oriented on the objectives of the study and trained according to the protocol. This training included health workers and government immunization staff—including district immunization officers, block and district medical officers, and CCHs. The trainings were provided in Nepali or Hindi and conducted at the block level with 108 participants attending across both districts.

## Results

### FPVC thermal performance

**Fig. 2:**
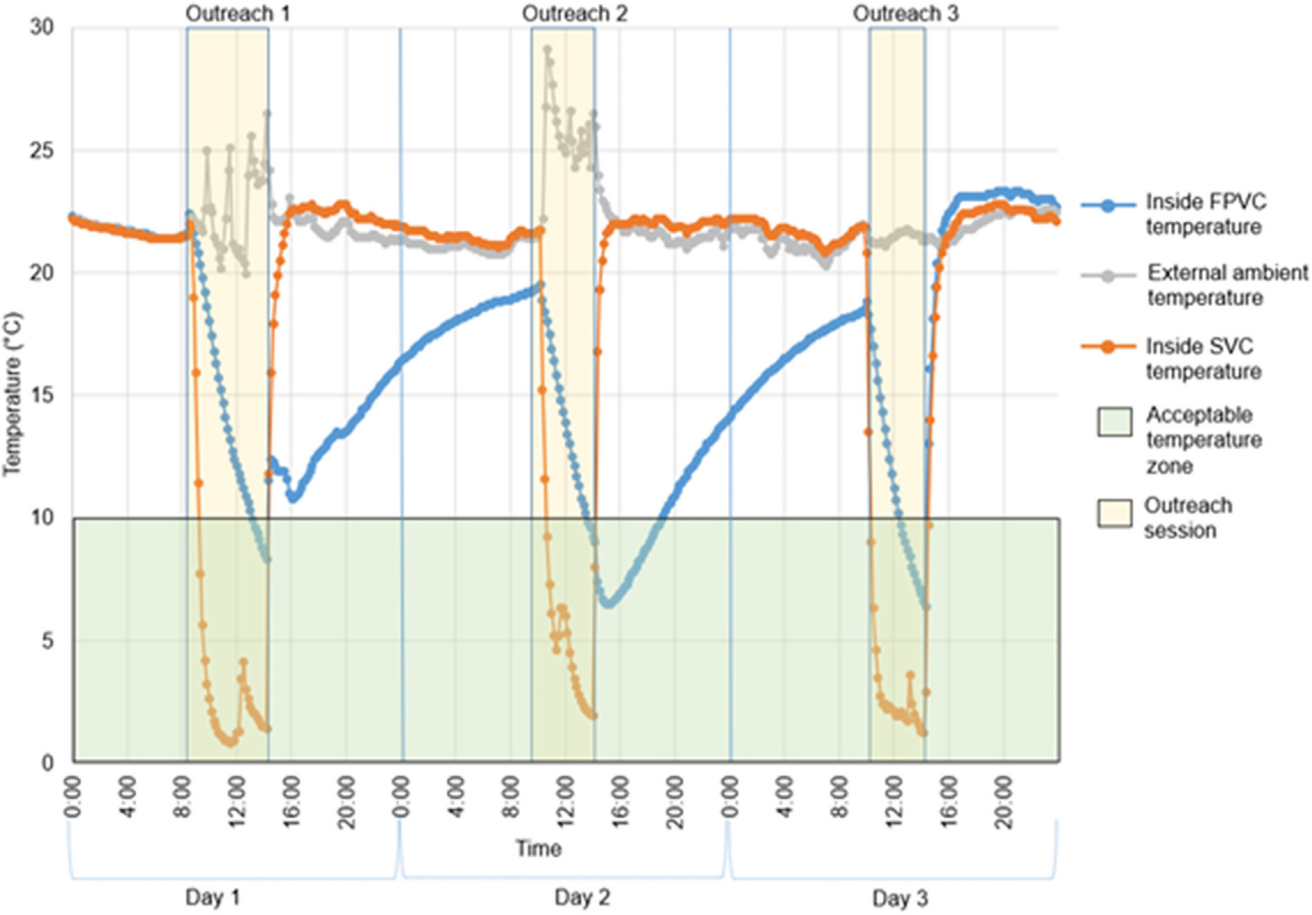
Example temperatures during three consecutive days of outreach.

Outreach sessions lasted one to three days. Fig. 2 shows temperatures during a typical three-day session, comparing an FPVC with fully frozen ice packs with an SVC with conditioned ice packs. The dips in the graph represent the start of the vaccine compartment cooling down, after the ice packs were placed in the carriers. The patterns show the effect of the FPVC barrier mechanism on slowing down the rapid drop in temperature experienced as soon as ice packs are introduced. It is at this point that freezing most often occurs.

#### Mean internal and mean kinetic temperature

Vaccines experience a range of temperatures inside carriers over time. Calculating MKT provides a single temperature value based on this range that can be used to determine the potency of vaccines as if they were left at a steady temperature. Although taking a simple average of temperatures often provides a similar result, MKT is a more rigorous metric for gauging the effect of higher temperatures on the thermal degradation of vaccines. MKT is influenced by time: the longer the ice packs remain in a carrier, the lower the MKT.

Table 1 shows the mean temperature and MKT for FPVCs in each block across all phases. The MKT is slightly higher than the mean temperature, reflecting the non-linear, increased thermal degradation caused by higher temperatures. The hilly blocks tended to have lower MKTs than the plains blocks, and the SVCs tended to have lower MKTs than the FPVCs.

**Table 1:** Internal temperatures by phase and block.

**District**	**Block**	**Terrain**	**FPVC mean temperature (°C)**	**FPVC mean kinetic temperature (°C)**	**SVC mean temperature (°C)**	**SVC mean kinetic temperature (°C)**
**Phase 1 – AOV**						
Sunsari	Harinagara	plains	19.5	20.8	6.8	9.3
Sunsari	Itahari	plains	19.0	20.6	7.5	10.9
Sunsari	Sitaganj	plains	17.7	19.8	5.6	9.4
Dhankuta	Dandabazar	hilly	9.4	10.8	5.0	7.0
Dhankuta	Pakhribas	hilly	5.3	6.7	4.4	5.0
**Phase 1a – Leff Trade**						
Sunsari	Harinagara	plains	20.7	22.9	10.9	16.0
Sunsari	Itahari	plains	14.8	17.0	6.3	12.2
Dhankuta	Dandabazar	hilly	7.0	8.0	6.7	9.3
Dhankuta	Pakhribas	hilly	6.3	7.5	5.8	8.2
**Phase 1a – Blowkings**						
Sunsari	Sitaganj	plains	14.5	16.9	5.4	7.6
Sunsari	Itahari	plains	13.9	16.8	5.8	7.5
Dhankuta	Dandabazar	hilly	9.8	12.3	5.8	7.5
Dhankuta	Pakhribas	hilly	6.8	8.2	5.8	7.5
**Phase 2 – all three FPVCs**						
Sunsari	Sitaganj	plains	9.8	10.4	N/A	N/A
Sunsari	Itahari	plains	9.6	10.4	N/A	N/A
Sunsari	Harinagara	plains	8.2	8.6	N/A	N/A
Dhankuta	Dandabazar	hilly	4.6	4.8	N/A	N/A
Dhankuta	Pakhribas	hilly	3.5	3.6	N/A	N/A

Abbreviations: FPVC, freeze-preventive vaccine carrier; N/A, not applicable; SVC, standard vaccine carrier.

**Fig. 3:**
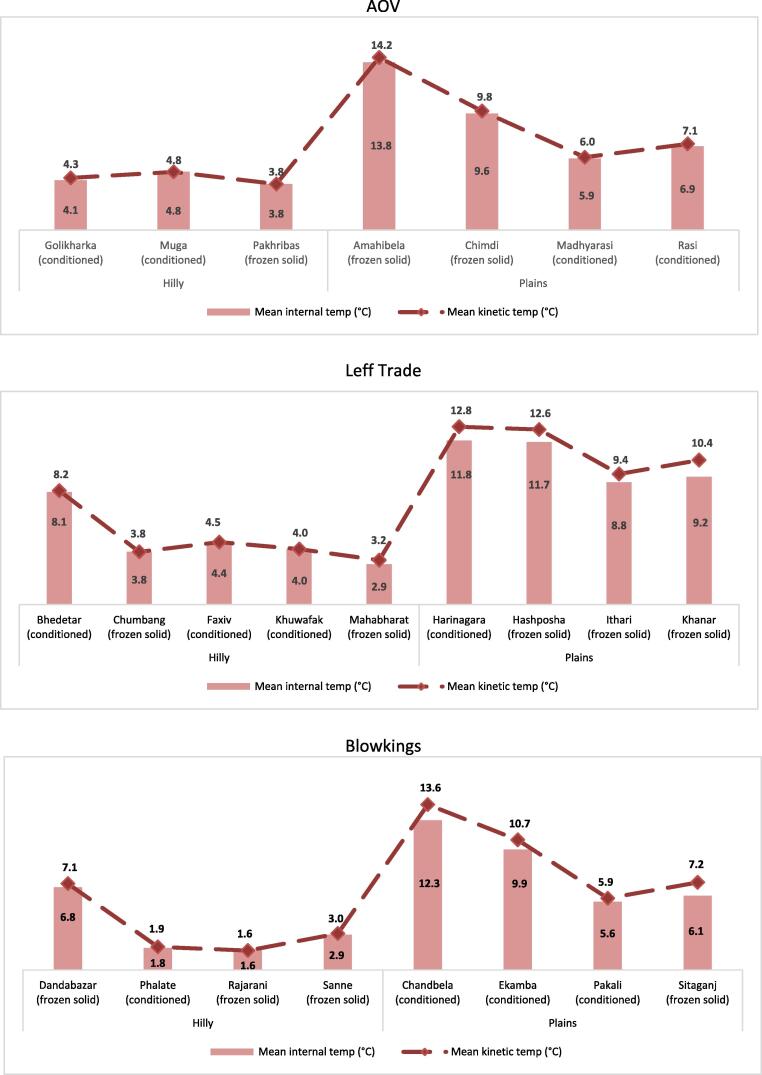
Mean internal temperature and mean kinetic temperature by health post and ice-pack state, phase 2.

The study also compared the MKT of blocks assigned frozen-solid ice packs and those assigned conditioned ice packs and found no significant difference (+7.2 °C and +7.0 °C, respectively, in phase 2). In phase 2, for blocks with frozen-solid ice packs, the highest mean temperature was 13.8 °C. The highest mean internal temperature with conditioned ice packs was 12.3 °C. Eighteen of 24 facilities (75%) maintained an MKT between 0 °C and +10 °C (Fig. 3).

#### Temperature excursions

In all phases, temperature readings were taken every 10 min while the vaccines were inside the carriers. In total, 3,618 h of temperature data were recorded. (Data were excluded when unavailable; when it was unclear if or at what time ice packs were placed in the carrier; and when the internal temperature was more than 0.5 °C above the ambient temperature, indicating unintentional placement outside the carrier. The latter occurred 24 times in phase 1 and 10 times in phase 1a.) Because passive vaccine carriers should maintain temperatures between 0 °C and +10 °C, internal temperature readings below or above this range were deemed excursions. In phase 1, low-temperature excursions (i.e., freezing temperatures) occurred in 2.7% of SVC sessions (Table 2). These freezing events lasted from 10 to 270 min at an average temperature range of –0.3 °C to –4.9 °C, and occurred most often in locations where health workers were assigned frozen-solid rather than conditioned ice packs. In phases 1 and 1a, there were no low-temperature excursions in the FPVCs (one was excluded; see note below Table 2). In phase 2, 0.2% of FPVC readings were below 0 °C, ranging from 0 °C to –0.4 °C. This represents nine sub-zero readings (in one session) at the Pakhribas HP. In phase 2, of the 925 high-temperature excursions in the FPVCs, 104 were in the hilly region and 821 were in the plains region; 547 were in carriers with frozen-solid ice packs, and 378 were in carriers with conditioned ice packs.

**Table 2:** Distribution of internal temperature readings by temperature category, phase, and carrier.

**Internal temperature**	**Number of readings**	**Percentage**	**Number of readings**	**Percentage**
**Phase 1**				
	**AOV FPVC**		**SVC**	
< 0 °C	0	0.0%	145	2.7%
0 °C to +10 °C	1,717	32.6%	4,190	78.9%
greater than 10 °C	3,555	67.4%	977	18.4%
Total readings	5,272		5,312	
**Phase 1a**				
	**Leff Trade FPVC**		**SVC**	
< 0 °C	*1	0.04%	5	0.2%
0 °C to +10 °C	1,633	57.5%	2,192	77.0%
greater than 10 °C	1,204	42.4%	647	22.7%
Total readings	2,838		2,844	
**Phase 1a**				
	**Blowkings FPVC**		**SVC**	
< 0 °C	0	0.0%	73	3.2%
0 °C to +10 °C	1,113	48.9%	1,720	76.4%
greater than 10 °C	1,165	51.1%	459	20.4%
Total readings	2,278		2,252	
**Phase 2**				
	**All FPVCs**		**SVC**	
< 0 °C	**9	0.2%	N/A	N/A
0 °C to +10 °C	2,822	75.1%	N/A	N/A
greater than 10 °C	925	***24.6%	N/A	N/A
Total readings	3,756		N/A	N/A

Abbreviations: FPVC, freeze-preventive vaccine carrier; N/A, not applicable; SVC, standard vaccine carrier.

* Previous research has shown that once carriers reach a steady temperature, they do not drop below freezing unless more ice packs are added. Given the time of day that this freeze event happened, it is postulated that after completing an outreach session, the health care worker placed the LogTag TRIX-8 in the freezer along with the ice packs. Therefore, this single freeze event was excluded from further analysis.

** These nine readings constitute one excursion at Pakhribas health post, representing 10% of the readings taken across all outreach sessions at that location. The freezing excursion was noted in an AOV carrier with frozen-solid ice packs at an average ambient temperature of 15 °C.

*** By carrier: 34% of AOV readings, 22.6% of Leff Trade readings, and 19.6% of Blowkings readings.

#### Ambient temperature analysis

**Fig. 4:**
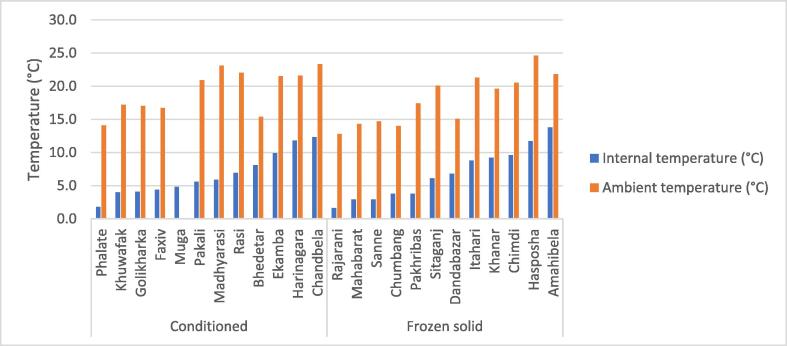
Mean internal temperature versus mean ambient temperature by health post, phase 2.

Per WHO PQS specifications, an FPVC must be able to maintain an internal temperature range of 0 °C to +10 °C at an ambient temperature range of +15 °C to +43 °C. During phase 1, higher ambient temperatures appeared to affect the MKTs of the AOVs to a greater extent than they affected the SVCs. In phase 1a, higher ambient temperatures appeared to raise the MKTs in the Leff Trade and Blowkings, but not in the SVCs. In phase 2, plotting the average ambient temperature against the mean internal temperature for each HP across all carrier brands studied shows some correlation between the temperature outside and inside the FPVCs (Fig. 4).

#### Cool-down time and rate

As per WHO PQS specifications, an FPVC must be capable of cooling from +43 °C to less than +10 °C in 8 h or less. To do so, a carrier must cool at an approximate average rate of 4 °C/hour or faster. Slower cool-down rates can still result in complete cool-downs if the starting temperature is below 43 °C. Table 3 lists the average cool-down times and rates by block and carrier in all phases. Average cool-down times and percentage of sessions in which the vaccine carrier successfully cooled down for the FPVCs ranged widely by block and phase. However, the average cool-down rate for each carrier type was relatively consistent.

**Table 3:** Average cool-down times and rates by block and carrier.

**Block**	**# of cool-downs**	**Avg. cool-down time (hour)**	**Avg. cool-down rate (°C/hour)**	**Reached < 10 °C (%)**	**# of cool-downs**	**Avg. cool-down time (hour)**	**Avg. cool-down rate (°C/hour)**	**Reached < 10 °C (%)**
**Phase 1**	**AOV FPVC**				**SVC**			
Dandabazar	38	3.65	2.9	84	33	0.51	32.1	97
Harinagara	26	6.29	2.6	15	15	0.70	44.1	100
Itahari	74	5.68	3.0	23	70	0.75	37.7	94
Pakhribas	18	2.92	3.0	100	25	0.47	28.3	100
Sitaganj	37	4.11	3.9	30	25	0.51	48.1	100
**Phase 1a**	**Leff Trade FPVC**				**SVC**			
Dandabazar	7	2.14	5.2	100	9	1.00	22.9	100
Harinagara	19	3.50	4.5	37	25	0.57	42.4	100
Itahari	26	3.12	5.6	85	16	0.40	40.3	100
Pakhribas	15	2.02	5.5	93	18	0.52	35.2	83
**Phase 1a**	**Blowkings FPVC**				**SVC**			
Dandabazar	14	1.91	4.5	64	11	0.83	36.4	100
Itahari	23	2.83	5.5	70	25	0.52	42.2	96
Pakhribas	14	1.86	5.5	100	17	0.41	28.5	94
Sitaganj	23	2.94	5.7	78	15	0.39	29.4	100
**Phase 2**	**All FPVCs**				**SVC**			
Dandabazar	12	1.15	3.2	100	N/A	N/A	N/A	N/A
Harinagara	7	3.12	3.3	100	N/A	N/A	N/A	N/A
Itahari	40	1.91	4.3	95	N/A	N/A	N/A	N/A
Pakhribas	6	1.39	4.8	100	N/A	N/A	N/A	N/A
Sitaganj	27	1.92	4.0	81	N/A	N/A	N/A	N/A

Abbreviations: FPVC, freeze-preventive vaccine carrier; SVC, standard vaccine carrier.

Table 4 shows the average cool-down times and rates for frozen-solid versus conditioned ice packs. In phase 2, the percentage of successful cool-downs was, on average, greater than 90%.

**Table 4:** Average cool-down times and rates by assigned ice-pack state and carrier.

**Ice-pack assignment**	**# of cool-downs**	Avg. cool-down time*(hours)	Avg. cool-down rate**(°C/hour)	**Reached < 10 °C (%)**	**# of cool-downs**	Avg. cool-down time*(hour)	Avg. cool-down rate**(°C/hour)	**Reached < 10 °C (%)**
**Phase 1**	**AOV FPVC**				**SVC**			
Conditioned	83	4.22	2.8	39	71	0.61	34.9	97
Frozen solid	110	4.02	4.0	45	97	0.62	39.1	97
**Phase 1a**	**Leff Trade FPVC**				**SVC**			
Conditioned	22	2.68	4.7	55	27	0.70	31.1	89
Frozen solid	45	2.75	5.5	84	41	0.50	41.6	100
**Phase 1a**	**Blowkings FPVC**				**SVC**			
Conditioned	37	2.38	5.5	81	32	0.40	28.9	97
Frozen solid	37	2.60	5.2	73	36	0.62	40.4	97
**Phase 2**	**All FPVCs**				**SVC**			
Conditioned	61	1.76	4.3	90	N/A	N/A	N/A	N/A
Frozen solid	31	2.07	3.5	97	N/A	N/A	N/A	N/A
**Phase 2**	**By brand**				**SVC**			
AOV FPVC	22	2.88	2.1	77	N/A	N/A	N/A	N/A
Leff Trade FPVC	39	1.71	4.1	97	N/A	N/A	N/A	N/A
Blowkings FPVC	31	1.49	5.2	97	N/A	N/A	N/A	N/A

Abbreviations: FPVC, freeze-preventive vaccine carrier; N/A, not applicable; SVC, standard vaccine carrier.

* Calculated if the internal temperature of the carrier started above 10 °C and cooled to below 10 °C.

** Calculated if the internal temperature of the carrier started above 10 °C and cooled, regardless of whether it cooled to below 10 °C.

#### Vaccine vial monitor life loss

Although the primary purpose of this study was to assess the ability of the FPVCs to prevent freezing temperatures from occurring, it is important to consider their ability to protect vaccines from heat exposure. VVMs are stickers attached to vaccine vials that change color when exposed to heat over time [10]. This color change, which serves as a proxy for the degradation of heat-sensitive vaccines, can be described through equations that depend on the category of VVM [11]. These equations estimate the percentage of vaccine life that has been lost due to exposure to different temperatures—referred to as VVM life loss. For reference, the most heat-sensitive vaccines, represented by VVM2, can remain at 20 °C for just 18 days before losing 100% life, while VVM7 vaccines can withstand 78 days at 20 °C, and VVM14 vaccines can withstand 156 days at 20 °C. Most Expanded Programme on Immunization vaccines are best represented by VVM14 or VVM30. To be conservative, we used the VVM2 equation to calculate a theoretical, worst-case VVM life loss. In phase 2 using real vaccines, the typical VVM life loss was just 0.25%, with the maximum remaining <1%. Results were consistent across blocks, and when we compared the assigned ice-pack state, there was little difference between sites assigned frozen-solid ice packs (0.22%) and those assigned conditioned ice packs (0.29%). Finally, we compared the average VVM life loss per session for each brand of carrier. The averages were quite close, with Blowkings at 0.21%, Leff Trade at 0.24%, and AOV at 0.31%.

### Electronic temperature monitors

In phases 1 and 1a, eight LogTag TRIX-8 recorders had errors or did not record internal temperatures. The devices that malfunctioned had all been placed in SVCs where high levels of condensation resulted in a pool of water at the bottom of the carrier. Though not clearly established, it is likely the water negatively impacted both the TRIX-8 and Trek 1.1 monitoring devices, so health workers began placing them in resealable plastic (Ziploc®) bags. In phase 2, only one TRIX-8 had errors and did not record ambient temperature. Yet the Trek 1.1 devices were sometimes replaced two or three times for the same HP due to functional issues. There were challenges both in updating the devices and the Parsyl mobile app with software upgrades. In addition, data could not be retrieved from four Trek 1.1 devices during the final data download and transfer process.

### Freezer performance

**Fig. 5:**
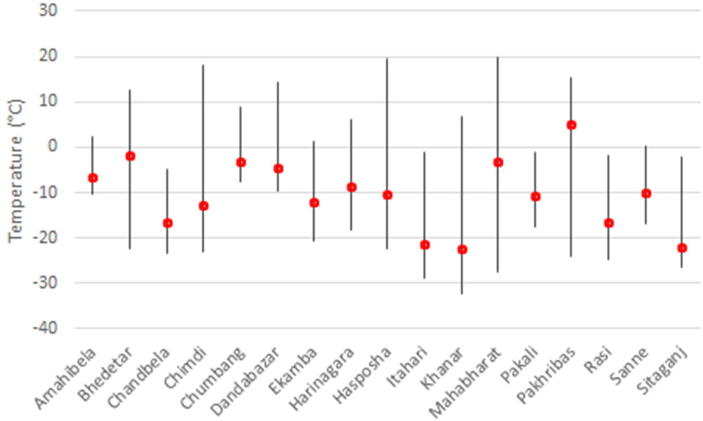
Mean and range of freezer temperatures by health post for phase 2.

The correct temperature range for freezers in the vaccine cold chain is –15 °C to –25 °C [12]. In phase 1, mean freezer temperatures ranged widely, from –24.6 °C to +10.6 °C. It is notable that the sites in this phase with the lowest mean freezer temperatures had the highest incidence of freezing temperatures in SVCs and that some freezers did not maintain freezing temperatures on average. In phase 1a, mean freezer temperatures ranged from –15.1 °C to +19.1 °C. In phase 2, mean freezer temperatures ranged from –22.4 °C to +5.1 °C, though most freezers had average temperatures between –16.6 °C and –1.9 °C. The coldest freezer in phase 2 still reached a maximum temperature of –5.0 °C, while the freezer with the highest maximum temperature (assuming the TRIX-8 was correctly placed in the freezer) reached 19.8 °C. Fig. 5 plots freezer performance for the duration of the phase.

### Acceptability and systems fit

The study used a baseline survey and in-depth interviews of health workers in all phases to collect data on the acceptability of the FPVCs. In all phases, health workers appreciated the freeze protection offered by the FPVCs and noted, based on VVM status, that the FPVCs did not inadvertently create a new problem of heat exposure. They also appreciated the potential for reduced wastage because no vaccine vial labels peeled off due to water damage, as they did in the SVCs. Health workers instructed to use frozen-solid ice packs felt that time was saved by not having to condition the ice packs. None of the facilities reported any major FPVC malfunctions. Size and weight of the FPVCs were the most pressing issues echoed by almost all health workers and health officials across the three carriers, though the SVCs traditionally used in the health system are shorter range than the FPVCs used in the study (thus smaller and lighter).

In phases 1 and 1a, health workers were asked to carry two vaccine carriers (FPVC along with SVC). In phase 1, there was some confusion because many health workers thought the AOV FPVC would always be transported with the SVC, requiring more space and staff. In addition to its size and weight, other AOV design issues (noted much less frequently) were difficulty in opening and closing the lid, difficultly removing ice packs when fully frozen, accidental tearing of the foam discs, and uncomfortable backpack and shoulder strap design.

Among the health workers using the Leff Trade in phase 1a, almost half preferred the SVC, and the other half preferred the Leff Trade over the SVC in its current condition. None of the health workers reported any breakage, and there was no reported condensation in the vaccine compartment. Most health workers said the Leff Trade took almost the same time or less than the SVC to clean. The backpacks were well received. ANMs shared that carrying the FPVC would be difficult without it. All the health workers said it was easy to insert and remove the ice packs. Almost all mentioned the Leff Trade had sufficient storage capacity, although two said their vaccines did not fit; these were health workers who had to carry a week’s supply of vaccines. Health workers gave similar feedback on the Blowkings in phase 1a. Most thought the foam discs were of good quality, though a few thought they were already showing signs of wear. Almost as many health workers preferred using the Blowkings to the SVC in its current condition. Suggestions for improvement included providing a thermometer and backpack.

In phase 2, health workers uniformly agreed that all three types of FPVCs enhanced safety, reduced vaccine wastage, and prevented vaccines from freezing, yet were larger and heavier than the SVCs. However, many did not find this to be a significant issue. Most health workers who mentioned size said their chief concern was the FPVCs would not fit on their scooters or bikes the way the SVCs do. All the health workers who mentioned weight as a significant issue were in the hilly areas. District-level health officials and block monitors stated that the size and weight of the FPVCs might be something to which health workers could adapt. Some ANMs mentioned that with time they might adjust if these devices were implemented and scaled up by the national MOHP. One health worker suggested that two sizes of FPVC should be developed: standard for regular sessions, and smaller for catchment areas with fewer beneficiaries. A few workers stated that the backpacks need more pockets, and one said padding should be added to the straps. ANMs, particularly in facilities using frozen-solid ice packs, pointed out that ice packs often expanded on freezing and were hard to insert into and remove from some FPVCs. Further, health workers noted that compared to the 0.4-liter ice packs used in the SVCs and Blowkings FPVCs, the 0.6-liter packs of the AOV and Leff Trade carriers were more difficult to fit into freezers. They also took longer to freeze, which was especially challenging for facilities experiencing frequent power cuts.

### Costs

Costs are presented in US dollars (US$). Using data from the logbooks, we obtained information from 54 outreach sessions. On average, HP staff vaccinated 15 children (range 1 to 72) and four pregnant women (range 0 to 19) during each outreach session. Table 5 shows the vaccines carried to these sessions, the price per dose, volume per dose, the number of doses per vial, and which of these vaccines are freeze sensitive. An average of 20 vials were carried to each session (range 2 to 79). The average value of the vaccines was $96.61. Of this, the average value of freeze-sensitive vaccines per session was approximately $70 (range $7 to $257). Thus, any one freezing incident during an outreach session could potentially damage about $70 worth of vaccine, on average.

**Table 5:** Types, average quantities, values, and volumes of vaccines taken to outreach sessions, phase 2.

	**BCG**	**OPV**	DTP*	PCV*	**MR**	**JE**	TD*		
Price per dose	$0.105	$0.13	$0.69	$3.05	$0.656	$0.41	$0.129		
Volume per dose	1.5044	0.8787	2.109	4.8	5.2514	2.9	2.122		
Doses per vial	20	20	10	2	10	5	10		
Freeze sensitive	No	No	Yes	Yes	No	No	Yes		
**Quantity of vials carried per outreach session**									
	Number of vials for each vaccine							***Total quantity***	
Average	2	2	2	9	2	2	1	***20***	
Minimum	0	0	0	1	0	0	1	***2***	
Maximum	11	7	10	30	8	9	4	***79***	
**Value of vaccines carried per outreach session**									
	Values for each vaccine (US$)							***Total value: all vaccines***	***Total value for the freeze-sensitive vaccines***
Average	$4.20	$5.20	$13.80	$54.90	$13.12	$4.10	$1.29	***$96.61***	***$69.99***
Minimum	$0.00	$0.00	$0.00	$6.10	$0.00	$0.00	$1.29	***$7.39***	***$7.39***
Maximum	$23.10	$18.20	$69.00	$183.00	$52.48	$18.45	$5.16	***$369.39***	***$257.16***
**Volume of vaccines carried per outreach session**									
	Volume for each vaccine in cm^3^							***Total volume in cm^3^***	***Total volume in liters***
Average	60.2	35.1	42.2	86.4	105.0	29.0	21.2	***379.2***	***0.379***
Minimum	0.0	0.0	0.0	9.6	0.0	0.0	21.2	***30.8***	***0.031***
Maximum	331.0	123.0	210.9	288.0	420.1	130.5	84.9	***1,588.4***	***1.588***

* Freeze-sensitive vaccines. Abbreviations: BCG, bacillus Calmette–Guérin; DTP, diphtheria-tetanus-pertussis; JE, Japanese encephalitis; MR, measles-rubella; OPV, oral poliovirus; PCV, pneumococcal conjugate vaccine; TD, tetanus-diphtheria.

We then calculated the benefit-cost ratio per health facility per year (Table 6). These calculations considered the minimum, average, and maximum value of freeze-sensitive vaccines that could potentially be prevented from freezing if FPVCs were used, as the benefit, and the annualized purchase price of the FPVC, as the cost. The price for an FPVC ranges from $39 to $55 depending on the manufacturer, with a median price of $45 [13], [14], [15]. When the median purchase price is divided over 5 or 10 years, the annualized price is $9 or $4.50, respectively. A benefit-cost ratio greater than 1 shows that the benefit outweighs the cost. The larger the benefit-cost ratio, the better the value for money. As shown in the table, all of the benefit-cost ratios are greater than 1 except when we use the minimum value ($7.39) of freeze-sensitive vaccines taken to a session and the 5-year annualized purchase price of the FPVC. However, even when we use the minimum value of freeze-sensitive vaccines taken to a session but annualize the FPVC purchase price over a 10-year period, the benefit outweighs the cost.

**Table 6:** Benefit-cost ratio of using freeze-preventive vaccine carriers per health facility per year.

	**Value**			**Variable in the calculation or formula**
**Cost**				
Median price of an FPVC	$45.00			C
Annualized price of an FPVC over 5 years	$9.00			C1 = C / 5
Annualized price of an FPVC over 10 years	$4.50			C2 = C / 10
**Assumption (based on study data)**				
Percentage of outreach sessions where vaccines are exposed to freezing temperatures	2.73%			P1
**Benefit**				
	Minimum	Average	Maximum	
Value of freeze-sensitive vaccines taken to an outreach session	$7.39	$69.99	$257.16	V
Number of outreach sessions held per month	3	4	4	S
Annual value of freeze-sensitive vaccines prevented from exposure to freezing during outreach sessions (taking into account the probability of vaccines being exposed to freezing temperatures in standard vaccine carriers at study sites)	$7.26	$91.70	$336.94	B = P1 × V × S × 12
**Benefit-cost ratios**				
Benefit-cost ratio (assuming 5-year useful life for the FPVC)	0.81	10.19	37.44	B / C1
Benefit-cost ratio (assuming 10-year useful life for the FPVC)	1.61	20.38	74.88	B / C2

Abbreviation: FPVC, freeze-preventive vaccine carrier.

## Discussion

There were no freezing temperatures in the FPVCs in phases 1 and 1a. As with many studies, knowing data were being collected may have positively influenced health worker practices. In phase 2, internal FPVC temperatures below 0 °C were registered in only one outreach session, in Pakhribas. At the time, Pakhribas was experiencing ambient temperatures below the PQS minimum for freeze prevention. (The mean ambient temperature for the 24 h prior to the start of the session was 11.5 °C.) There were no SVCs used in phase 2, so there is no directly relatable data indicating how an SVC would have performed at the same time. PQS testing for carriers only requires them to show freeze prevention at a minimum rated ambient temperature of 15 °C. Yet FPVCs are well suited to decrease risk in scenarios like winter vaccination sessions, where low ambient temperatures pose a greater risk of freezing the vaccines.

Data on freezer temperatures point to wide variations in freezer performance that affected both ice-pack freezing and the ability of ice packs to maintain cold temperatures. Eleven of the study freezers were not PQS prequalified. In remote settings, freezer temperatures rarely reached below –10 °C and sometimes were considerably warmer. Yet freezers that consistently maintain cooler temperatures may also result in more fully frozen ice packs and increased incidence of vaccine freezing. Freezers were more consistent at supplying sub-zero temperatures in phase 2. This could be due to the lower ambient temperatures, which would keep the freezer cooler when the door was opened or during a power loss. It may also be that access to grid power is more consistent in winter.

The MKTs inside the FPVCs were substantially higher than in the SVCs. Throughout the study, the hilly blocks were able to maintain MKTs within the range of 0 °C to +10 °C more often than the plains blocks, which might be due to the lower ambient temperatures in the hilly blocks. Several factors are believed to have contributed. First, in the hilly blocks, more of the HPs than in the plains blocks placed their ice packs in the carriers the day before some or all of the outreach sessions due to logistical necessity. By the time these sessions began, the carriers were already cool. Conversely, not having a freezer on-site likely prevented some sites in both districts from actually using frozen-solid ice packs, as assigned, and warmer-than-expected freezer temperatures may have preconditioned some of the ice packs. Also, because frozen-solid ice packs can bulge, health workers may have had to let them condition for longer than recommended in order to fit them into the FPVC ice-pack compartments. Study results point to the same factors for why FPVC cool-down times were more variable than anticipated. Yet during actual use in phase 2, average cool-down times were <4 h across all vaccine carrier brands and ice-pack states, and more than 90% of carriers cooled to below 10 °C by the time the vaccination session ended.

Calculating VVM life loss showed that exposure to temperatures above 10 °C in the FPVCs had a negligible effect on vaccine potency. The session with the greatest VVM life loss, at 0.95%, occurred when the vaccines had remained in the carrier for multiple days. Over the course of the study, the average VVM life loss per session—including all HPs, conditioning states, and manufacturers—was just 0.25%. With the exception of the one freezing temperature event at Pakhribas, the FPVCs were successful in both preventing exposure to sub-zero temperatures and guarding against heat degradation.

FPVC selection criteria should consider storage capacity, need for short- versus long-range carriers, as well as distance and means of transport for the FPVC. This study included districts in hilly and plains settings, with the intention to evaluate the acceptability and systems fit in varying environments of use, including remote areas. However, outreach visits lasted consistently <8 h even in the hilly areas. This length of outreach is better suited for short-range carriers, regardless of terrain. Also, the smaller-capacity carriers were generally sufficient for the loads (on average, 0.38 L of vaccine and diluent) required in these areas of Nepal. The exception is remote areas lacking refrigeration, where carriers must be kept in the field for several days before changing ice packs.

### Limitations

Data gaps occurred for several reasons, including language barriers, inconsistent terminology, inexact time recordings, and LogTag TRIX-8 malfunctions from excessive exposure to water in SVCs (phases 1 and 1a). For the Parsyl Trek 1.1, challenges in data transfer were likely due to mobile app issues or phone connectivity rather than the hardware itself. Also, data were analyzed for each vaccine carrier type assuming the category of ice pack (frozen solid or conditioned) assigned to each site was indicative of the true state of the ice packs. The frozen-solid ice packs assigned to the HPs with no freezer may have been inadvertently conditioned during transport. Regarding freezer performance, the data were trimmed to remove ambient temperature readings at the beginning and end of each recording period, as well as temperature spikes that were unlikely to occur if the LogTags had remained in the freezer. This was done to ensure greater accuracy; however, there is a risk that some legitimate readings may have been excluded, or that some incorrect readings may have remained. Also, because freezer data were measured 24 h prior to the session, we cannot be certain how this influenced ice-pack freezing, as we do not know when the ice packs were placed in the freezers. Thus, discussions of the relationship between freezer performance and carrier performance are limited to noting generalized trends.

## Conclusions

By keeping vaccines within an optimal temperature range, the vaccine carriers used in this study averted a potential loss in potency of freeze-sensitive vaccines and, important for immunization budgets, loss in dollar value of vaccines. Study results validate that long-range FPVCs are a viable means of preventing freezing temperatures in outreach vaccination settings and are a good value for money. The study also reinforced how critical it is to verify the context of use prior to introduction of any new technology. Cool-down times for the FPVCs were more variable than laboratory testing predicted. This appears to have been driven primarily by higher-than-expected variations in freezer performance. More research is needed to understand to what extent this is due to the quality of the freezers, availability of consistent power, or other factors. As new, highly efficient water pack freezers are introduced worldwide, there may be an inadvertent, increased risk of vaccine freezing in SVCs. This study’s authors urge global immunization supply chain entities to include recommendations for countries to procure FPVCs, retrain health workers on conditioning ice packs, or consider using cool water packs in SVCs instead of ice packs. Immunization programs using FPVCs will similarly have to weigh the risk of vaccines potentially being above the recommended 0 °C to +10 °C range for several hours as the device cools. The FPVCs included in this study met their performance objectives, and users appreciated their benefits; however, most health workers did not like the increased size and weight of the long-range FPVCs in contrast to the smaller, lighter SVCs. Further efforts to design FPVCs should place a high priority on minimizing carrier size and weight.

## Data availability

Data presented in this article are available from the online repository *Mendeley Data* under the title, “Long-range freeze-preventive vaccine carriers in Nepal: thermal data” with digital object identifier https://doi.org/10.17632/j277dvs69g.1 [16].

## Declaration of Competing Interest

The authors declare that they have no known competing financial interests or personal relationships that could have appeared to influence the work reported in this paper.
